# The Effects of *Thymus* *Caramanicus* Jalas Extract and Its Main Constituent Carvacrol Against Cisplatin-Induced Nephrotoxicity in Mice

**DOI:** 10.5812/ijpr-140212

**Published:** 2024-07-03

**Authors:** Amin Hassanshahi, Ayat Kaeedi, Mohammad Reza Rahmani, Jalal Hassanshahi

**Affiliations:** 1 Department of Physiology, School of Medicine, Bam University of Medical Sciences, Bam, Iran; 2 Physiology-Pharmacology Research Center, Research Institute of Basic Medical Sciences, Rafsanjan University of Medical Sciences, Rafsanjan, Iran; 3 Department of Physiology and Pharmacology, School of Medicine, Rafsanjan University of Medical Sciences, Rafsanjan, Iran

**Keywords:** Cisplatin, Nephrotoxicity, *Thymus Caramanicus* Jalas, Anti-inflammatory Effect, Antioxidant, Apoptosis

## Abstract

**Background:**

Cisplatin, an anti-cancer chemotherapy drug, has nephrotoxic effects. *Thymus caramanicus* Jalas (TCJ) has antioxidant effects due to its main components.

**Objectives:**

In the current research, we assessed the impact of TCJ extract and its main compound on cisplatin-induced nephrotoxicity in mice.

**Methods:**

Forty-two male mice were used in the study. Depending on their group, the animals received saline, carvacrol (10 mg/kg), or TCJ extract (50, 100, and 150 mg/kg) for 10 days. On the fifth day, mice received cisplatin (7.5 mg/kg, i.p.). After 10 days, serum creatinine (Cr) and blood urea nitrogen (BUN) levels were measured. Additionally, malondialdehyde (MDA) and glutathione (GSH) contents, as well as the activity levels of superoxide dismutase (SOD), catalase, and glutathione peroxidase (GPx), and total antioxidant capacity (TAC) were measured in the kidney tissues. The western blotting method was used to determine the kidney's expression of cleaved caspase-3, Bax, Bcl-2, nuclear factor kappa-B (NF-κB), and tumor necrosis factor-alpha (TNF-α). Kidney tissue damage score (KTDS) was assessed using the hematoxylin-eosin (H&E) staining method.

**Results:**

Cisplatin significantly increased serum Cr, KTDS, MDA, BUN levels, NF-κB, TNF-α, cleaved caspase-3, and Bax protein expression in the cisplatin group compared to the control group (P < 0.01). Additionally, cisplatin significantly decreased the kidney tissue's TAC and GSH content, activity levels of SOD, catalase, and GPx indicators, and expression of Bcl-2 protein (P < 0.05). TCJ and carvacrol significantly ameliorated these indicators in the cisplatin + TCJ (150 mg/kg) and cisplatin + carvacrol (10 mg/kg) groups compared to the cisplatin group (P < 0.05).

**Conclusions:**

TCJ (150 mg/kg) and its main component, carvacrol, could somewhat reduce cisplatin-induced nephrotoxicity through their anti-inflammatory, antioxidant, and anti-apoptotic effects.

## 1. Background

Cisplatin, one of the alkylating agents, is a cell-cycle non-specific drug used in cancer treatment (1). Cisplatin-induced nephrotoxicity is the most critical side effect, limiting its use in treatment (2). Several mechanisms are involved in cisplatin-induced nephrotoxicity, the most important of which are the generation of reactive oxygen species (ROS) and tumor necrosis factor-α (TNF-α) (2, 3). Reactive oxygen species causes lipid peroxidation, leading to acute kidney injury (4). Additionally, cisplatin increases inflammation via the TNF-α-induced nuclear factor-kappa B (NF-κB) activation signaling pathway. Studies have shown that cisplatin induces oxidative stress in kidney tissue (5) and elevates serum creatinine (Cr) and blood urea nitrogen (BUN) levels (6). Furthermore, cisplatin reduces the activity levels of glutathione peroxidase (GPx) and superoxide dismutase (SOD) in kidney tissue, resulting in an increased lipid peroxidation index of malondialdehyde (MDA) (7). Research has reported that exogenous antioxidants can mitigate oxidative stress and apoptosis during cisplatin-induced renal injury (7). Administration of cisplatin in vivo at a nephrotoxic dose significantly increases kidney necrosis and apoptosis (8). The ability of cisplatin to trigger the intrinsic mitochondrial pathway of apoptosis is well-supported by available data (9). Exposure of renal epithelial cells to cisplatin leads to Bax translocation into mitochondria and caspase activation (10). The cell death protease caspase is critical for the execution of apoptosis during cisplatin-induced tubular epithelial cell death.

Due to the high side effects of chemical drugs, researchers have increased their interest in medicinal plants and the use of active compounds in treating diseases (11). In recent years, numerous herbs have been used to treat kidney disorders, and many of these herbal substances' antioxidant properties have been identified (11, 12). *Thymus caramanicus* Jalas (TCJ) is one such herb with antioxidant properties (13). This plant belongs to the *Lamiaceae* family; several family members are used as medicines to treat various diseases and originated in the Mediterranean region (14). These plants are mainly aromatic, evergreen, durable, shrubby, and have woody roots, commonly found in calcareous soils and grasslands throughout Europe and Asia (14). Various studies have reported antioxidant (15), antinociceptive/anti-inflammatory (16), antiapoptotic (14), and antitumor (17) effects of this plant. The healing effects of TCJ are mainly attributed to its polyphenols (13). Carvacrol, the main compound of TCJ, is known to be one of the most potent polyphenols (15). Carvacrol can attenuate acute kidney injury induced by cisplatin by suppressing oxidative stress, inflammation, and apoptosis (18). Carvacrol exhibits antioxidant activity in both in-vitro and in-vivo conditions (19, 20).

## 2. Objectives

Since cisplatin weakens the antioxidant system in the kidneys and leads to nephrotoxicity, and due to the TCJ biological effects, this study's objective was to ascertain the effects of TCJ extract and its main constituent carvacrol against cisplatin-induced nephrotoxicity in mice.

## 3. Methods

### 3.1. Plant Material and Preparation of its Extract

The aerial portion of TCJ was collected in the mountains of Kerman Province, Iran. The herbarium specimen was stored in the herbarium of the Department of Phytomedicine, Rafsanjan Medical University (Number: 97.1.1, Rafsanjan, Iran). A botanist confirmed the identification of the medicinal plant. *Thymus caramanicus* Jalas leaves were dried in direct sunlight, and their powder was prepared using a laboratory blender. Two hundred fifty grams of plant leaf powder was placed into a 2-liter bottle. Subsequently, a 1.5-liter mixture of water and 96% ethanol (in a 20:80 ratio) was added. The suspension was extracted for 4 hours (3 times). Finally, the TCJ extract was dried in a rotary evaporator (14).

### 3.2. Gas Chromatography-Mass Spectrometric Analysis (GC–MS)

Gas chromatography-mass spectrometry (GC-MS) analysis of TCJ extracts was performed using an Agilent 7890 GC-MS system 'JMS 700, USA'. It features an HP DB-5 capillary column with a 0.25 μm film thickness, 30 m × 0.25 mm ID, and an ionization potential energy of 70 eV. These specifications were used to analyze TCJ-extracted compounds (15).

### 3.3. Animal

The current study was conducted on 42 male mice weighing 30 ± 3 g. Every four mice were kept in a cage under controlled temperature and light conditions (12 h light/dark cycle, 23 ± 1°C) with free access to water and food. This study was conducted according to the protocols and guidelines recommended for the maintenance, care, and use of laboratory animals as revised in 2010 (National Institutes of Health Publication No. 85 - 23). Additionally, the Ethics Committee of Rafsanjan Medical University approved this study (ethical code: IR.RUMS.REC.1398.133).

### 3.4. Experimental Groups

In this experimental study, 42 male mice were randomly divided into 7 groups (n = 6), as listed below:

(1) Control group (vehicle): Animals received the vehicle (1% tween 80 in normal saline) at a dose of 1 mL/kg for 10 days by gavage (21).

(2) *Thymus caramanicus* Jalas (150 mg/kg) group: Animals received TCJ extract at 150 mg/kg (the highest dose used in this study) daily by gavage for 10 days.

(3) Cisplatin group: Animals received the vehicle at a dose of 1 mL/kg for 10 days by gavage. Additionally, on day 5, cisplatin was administered as a single intraperitoneal (i.p.) dose of 7.5 mg/kg.

(4) Cisplatin + TCJ (50 mg/kg) group: Animals received TCJ extract dissolved in 1% tween 80 in normal saline at a dose of 50 mg/kg for 10 days by oral gavage. Additionally, on day 5, they received cisplatin (7.5 mg/kg, i.p.).

(5) Cisplatin + TCJ (100 mg/kg) group: Animals received TCJ extract dissolved in 1% tween 80 in normal saline at a dose of 100 mg/kg for 10 days by oral gavage. Additionally, on day 5, they received cisplatin (7.5 mg/kg, i.p.).

(6) Cisplatin + TCJ (150 mg/kg) group: Animals received TCJ extract dissolved in 1% tween 80 in normal saline at a dose of 150 mg/kg for 10 days by oral gavage. Additionally, on day 5, they received cisplatin (7.5 mg/kg, i.p.).

(7) Cisplatin + Carvacrol (10 mg/kg) group: Animals received carvacrol dissolved in 1% tween 80 in normal saline at a dose of 10 mg/kg for 10 days by oral gavage (21). Additionally, on day 5, they received cisplatin (7.5 mg/kg, i.p.).

### 3.5. Study Protocol

In this study, animals received solutions containing 1% tween 80 in normal saline, carvacrol (10 mg/kg), or TCJ extract (50, 100, and 150 mg/kg) depending on their group for 10 days (14, 18). The cisplatin (Sigma, USA), TCJ extract, and carvacrol (Sigma, USA) were dissolved in 1% tween 80 in a standard saline solution (21). Additionally, on day 5, mice received cisplatin (single dose, 7.5 mg/kg, i.p.) (7). At the end of the tenth day, the animals were anesthetized with urethane (1.7 g/kg, i.p.) (Sigma, USA), followed by measurements of the various parameters described below.

#### 3.5.1. Serum Creatinine and BUN Levels Measurement

After anesthetizing the animals with urethane (1.7 g/kg, i.p.) (Sigma, USA), the paw pinch reflex was checked in each mouse (4). Blood samples were then taken from the arteries in the corners of the eyes, centrifuged to separate the serum, and subsequently measured for BUN and Cr levels using a BUN and Cr assay kit (Pars Azmoon Co., Iran) (22).

#### 3.5.2. Renal Tissue Preparation for Molecular Analysis

After blood collection, the animals' heads were severed with a guillotine while under deep anesthesia. Their kidneys were then immediately removed, and one kidney was frozen in liquid nitrogen. The kidney tissue was isolated and homogenized in ice-cooled RIPA buffer containing protease inhibitors [1 mM EDTA, 0.1% sodium dodecyl sulfate, 10 mM Tris–HCl at pH 7.4, 0.1% Na deoxycholate, 1% NP-40 with a protease inhibitor cocktail (2.5 μg/mL of leupeptin, 10 μg/mL of aprotinin), 1 mM sodium orthovanadate, and 1 mM phenylmethylsulfonyl fluoride] (23). Kidney tissue samples were frozen in a liquid nitrogen tank. The tissue samples were then homogenized and centrifuged for 20 minutes (14000 rpm at 4°C), and the supernatant was collected. The Bradford method was used to determine protein concentration in the supernatant (4, 23).

#### 3.5.3. Oxidative Stress Parameters Measurement

Some of the oxidative stress indices [MDA and glutathione (GSH) content, as well as the activity levels of catalase, GPx, SOD, and TAC] were measured in kidney tissue using a relevant commercial kit (ZellBio, Germany) (24-26).

#### 3.5.4. Western Blot Analysisn

Western blotting was used to analyze the protein expression levels of inflammatory markers (NF-κB and TNF-α) and apoptotic markers (cleaved caspase-3, Bax, and Bcl-2) in renal tissues (4, 27). Harvested protein samples were separated by electrophoresis on a 12.5% polyacrylamide gel and transferred to a nitrocellulose membrane. Each membrane was incubated overnight at 4°C in Tris-buffered saline with Tween 20 (0.1% Tween 20, 20 mM Tris-HCl, 150 mM NaCl) containing 5% non-fat milk, pH 7.4. Subsequently, the nitrocellulose membranes were incubated with monoclonal rabbit anti-TNF-α (Abcam, USA, EPR19147) (1: 1000), monoclonal rabbit anti-NF-κB (Abcam, USA, EP2294Y) (1: 1000), monoclonal rabbit anti-Bax (Abcam, USA, ab173026) (1: 1000), monoclonal rabbit anti-caspase-3 (Abcam, USA, ab184787) (1: 1000), and rabbit polyclonal anti-Bcl-2 (Abcam, USA, ab196495) (1: 1000) antibodies for three hours at room temperature. Each antibody was diluted using the blocking buffer. Blots were then washed three times with Tween 20 and incubated with an anti-rabbit horseradish peroxidase secondary antibody (Abcam, USA) (1: 5000) for one hour at room temperature. Blot detection was done using an enhanced chemiluminescence method. Finally, ImageJ software was used for band densitometry. Beta-actin immunoblotting (Abcam, USA) (1: 5000) was used as a loading control. The densitometric ratio of each protein band was compared to the beta-actin band for each sample (23, 28).

#### 3.5.5. Tissue Staining

The other kidney was embedded in 10% formaldehyde buffer, and histological examinations of kidney tissue were performed using the hematoxylin-eosin (H&E) staining method. In brief, all histopathological images were captured at the same resolution using a light microscope (Nikon, Japan). Then, an expert pathologist, who was blinded to the study procedures, analyzed the images using ImageJ software. Based on the severity of kidney tissue injury, a score ranging from 0 to 3 was assigned (0 - 0.5 = normal, no injury observed; 1 = minor; 2 = moderate; 3 = severe). Previous studies used several indices to assess the severity of kidney tissue injury, including tubular atrophy, tubular dilatation, tubular cell vacuolization, casts, interstitial infiltrates, and edema. Initially, a separate score of 0 to 3 was assigned to each index based on the severity of the injury, and then the sum of the scores was considered the kidney tissue damage score (KTDS) in a mouse (4, 11).

### 3.6. Statistical Analysis

GraphPad Prism software (version 6) was used for data analysis. All data were presented as mean ± standard deviation (SD). After confirming the normality of the frequency distribution of the collected data using the Shapiro-Wilk test (P > 0.05) and the equality of variance of the groups by Levene's test (P > 0.05), the intergroup differences were evaluated using one-way ANOVA and the Tukey post hoc test, with a significance level of 0.05 for all tests.

## 4. Results

### 4.1. Phytochemical Analysis of Thymus caramanicus Jalas Extract

**Figure 1.**
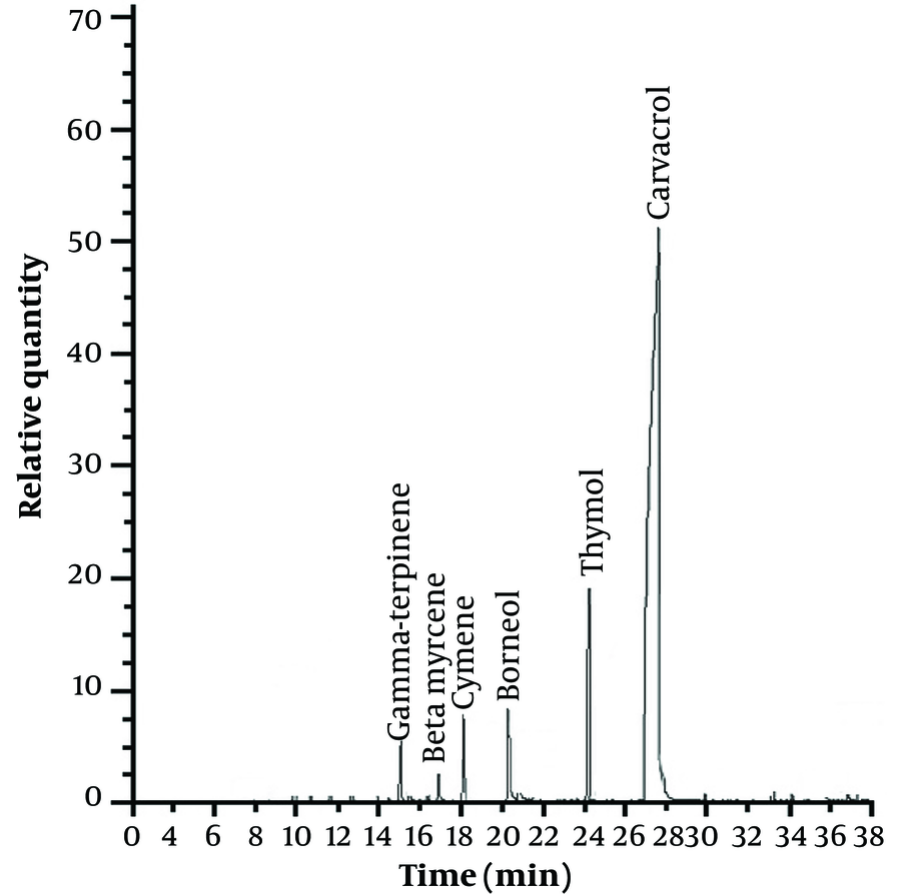
Chromatography of *Thymus caramanicus* Jalas extracts was analyzed by gas chromatography-mass spectrometry.

Analysis of the TCJ extract by gas chromatography and mass spectrometry showed that carvacrol (52.11%), thymol (19.43%), beta-myrcene (2.11%), borneol (6.20%), cymene (5.95%), and gamma-terpinene (4.96%) were some of the compounds in the extract (Figure 1). 

### 4.2. The Effect of Thymus Caramanicus Jalas and Carvacrol on Serum Creatinine and Blood Urea Nitrogen Levels

**Figure 2.**
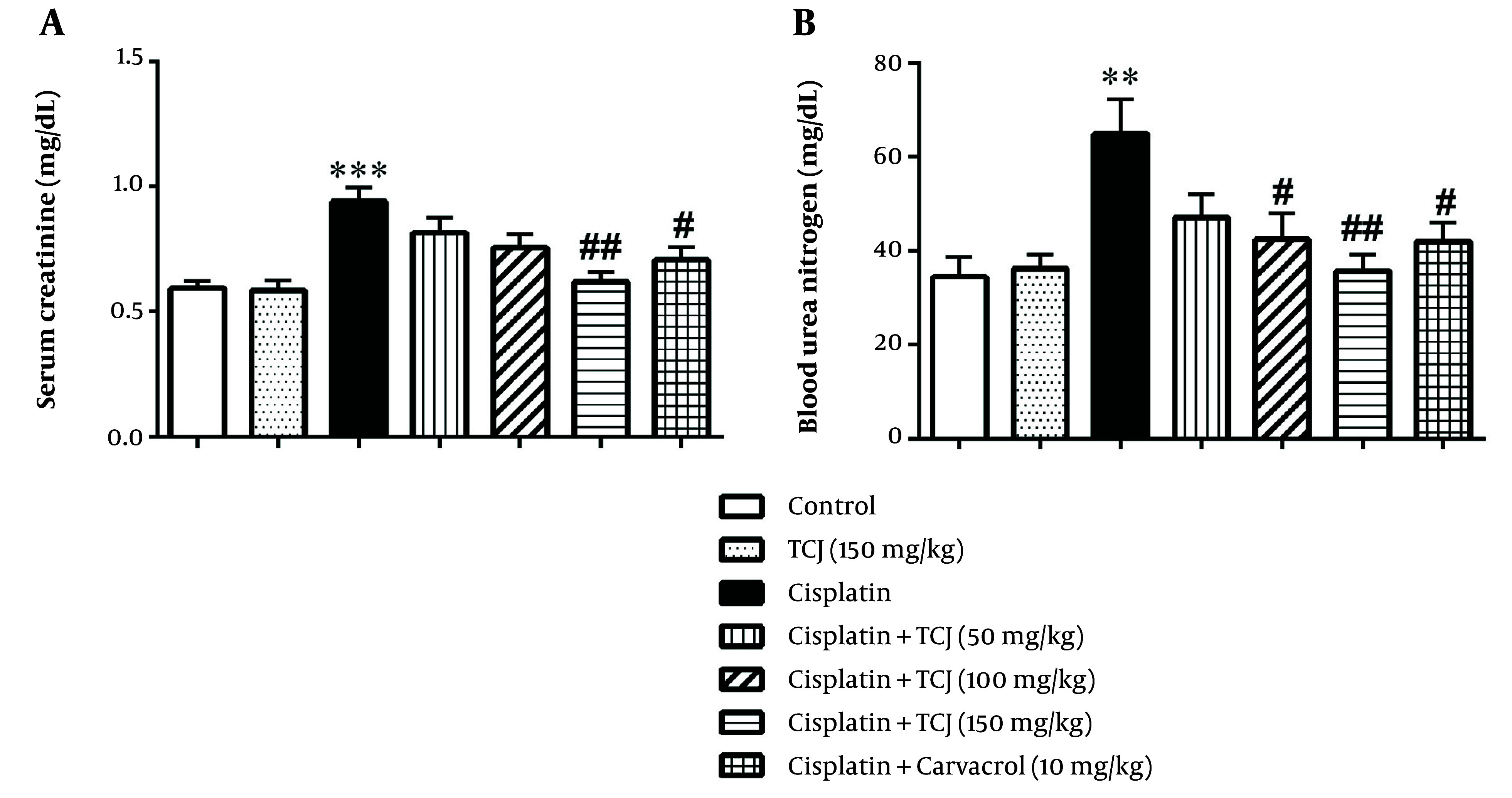
The serum creatinine and blood urea nitrogen levels in all groups at the end of the experiment. Each value is presented as mean ± SD. N = 6/group. ٭٭P < 0.01, and ٭٭٭P < 0.001 compared to the control. #P < 0.05, and ##P < 0.01 compared to the cisplatin group. TCJ: *Thymus caramanicus* jalas.

The results showed that cisplatin significantly increased Cr and BUN levels in the cisplatin-treated mice compared to the control group (P < 0.001 and P < 0.01 respectively, Figure 2). Moreover, TCJ extract (150 mg/kg) and carvacrol (10 mg/kg) significantly decreased BUN and Cr levels in the cisplatin + TCJ (150 mg/kg) and cisplatin + carvacrol (10 mg/kg) groups compared to the cisplatin group (P < 0.01 and P < 0.05 respectively) (Figure 2). Additionally, TCJ extract (100 mg/kg) significantly ameliorated the BUN level in the cisplatin + TCJ (100 mg/kg) group compared to the cisplatin-treated mice (P < 0.05, Figure 2). 

### 4.3. The Effect of Thymus Caramanicus Jalas and Carvacrol on Kidney Tissues' Oxidative Stress Parameters

According to Table 1, the MDA concentration, an oxidative stress marker, significantly increased in the kidney tissue of cisplatin-administered mice compared to the control group (P < 0.05). *Thymus caramanicus* jalas. (150 mg/kg) significantly decreased the MDA level in the kidney tissue of the cisplatin + TCJ (150 mg/kg) group compared to the cisplatin-administered mice (P < 0.05, Table 1). 

**Table 1.** The oxidative stress parameters (MDA and TAC levels, GSH content, SOD, catalase and GPx activity levels) in the kidney tissues at the end of the experiment ^a, b^

Group	MDA (nM/mg Protein)	Catalase (U/mg Protein)	GPx (mU/mg Protein)	GSH (nM/mg Protein)	SOD (U/mg Protein)	TAC (mmol/L)
**Control**	4.7 ± 1.64	81.4 ± 5.85	207.0 ± 33.70	39.5 ± 3.51	43.5 ± 6.47	1.3 ± 0.12
**TCJ (150 mg/kg) **	5.1 ± 1.74	78.1 ± 9.91	203.3 ± 37.91	39.6 ± 4.50	42.7 ± 6.47	1.2 ± 0.13
**Cisplatin**	8.2 ± 1.40 ^c^	60.5 ± 8.38 ^d^	141.7 ± 32.60 ^c^	27.8 ± 5.12 ^c^	28.5 ± 4.80 ^d^	0.93 ± 0.16 ^c^
**Cisplatin + TCJ (50 mg/kg) **	6.6 ± 1.87	56.7 ± 8.50	160.3 ± 28.88	31.6 ± 5.19	33.7 ± 5.16	0.98 ± 0.15
**Cisplatin + TCJ (100 mg/kg)**	5.7 ± 1.65	66.7 ± 11.13	192.8 ± 27.06	32.1 ± 5.20	35.2 ± 4.95	1.1 ± 0.17
**Cisplatin + TCJ (150 mg/kg) **	4.7 ± 1.40 ^e^	77.7 ± 7.84 ^e^	206.7 ± 20.61 ^e^	36.7 ± 3.88 ^e^	40.3 ± 7.36 ^e^	1.2 ± 0.12 ^e^
**Cisplatin + Carvacrol (10 mg/kg)**	6.1 ± 1.60	67.0 ± 11.45	203.7 ± 34.54 ^e^	35.2 ± 3.25	39.3 ± 5.88 ^e^	1.1 ± 0.23

Abbreviation: TCJ, *Thymus caramanicus* jalas; MDA, malondialdehyde; GPx, glutathione peroxidase; GSH, glutathione; SOD, superoxide dismutase; TAC, total antioxidant capacity.

^a^ Values are expressed as mean ± SD.

^b^ n = 6/group.

^c^ P ˂ 0.0.

^d^ P ˂ 0.01 compared to the control.

^e^ P ˂ 0.05 compared to the cisplatin group.

As shown in Table 1, cisplatin significantly decreased the GSH content (P < 0.05), TAC level (P < 0.05), SOD (P < 0.01), catalase (P < 0.01), and GPx (P < 0.05) activity levels in the kidney tissue of cisplatin-administered mice compared to the control group. Moreover, administration of TCJ (150 mg/kg) significantly increased the TAC level, GSH content, and catalase, GPx, and SOD activity levels in the kidney tissue of cisplatin + TCJ (150 mg/kg) treated mice compared to the cisplatin-administered mice (P < 0.05, Table 1). Furthermore, carvacrol significantly increased the GPx and SOD activity levels in the renal tissue of the cisplatin + carvacrol (10 mg/kg) group compared to the cisplatin-administered mice (P < 0.05, Table 1). However, carvacrol did not alter the MDA, catalase, and TAC indicators in the kidney tissue of the carvacrol-treated mice that received cisplatin (10 mg/kg).

### 4.4. The Effect of Thymus Caramanicus Jalas and Carvacrol on Kidney Tissues Inflammatory Parameters

**Figure 3.**
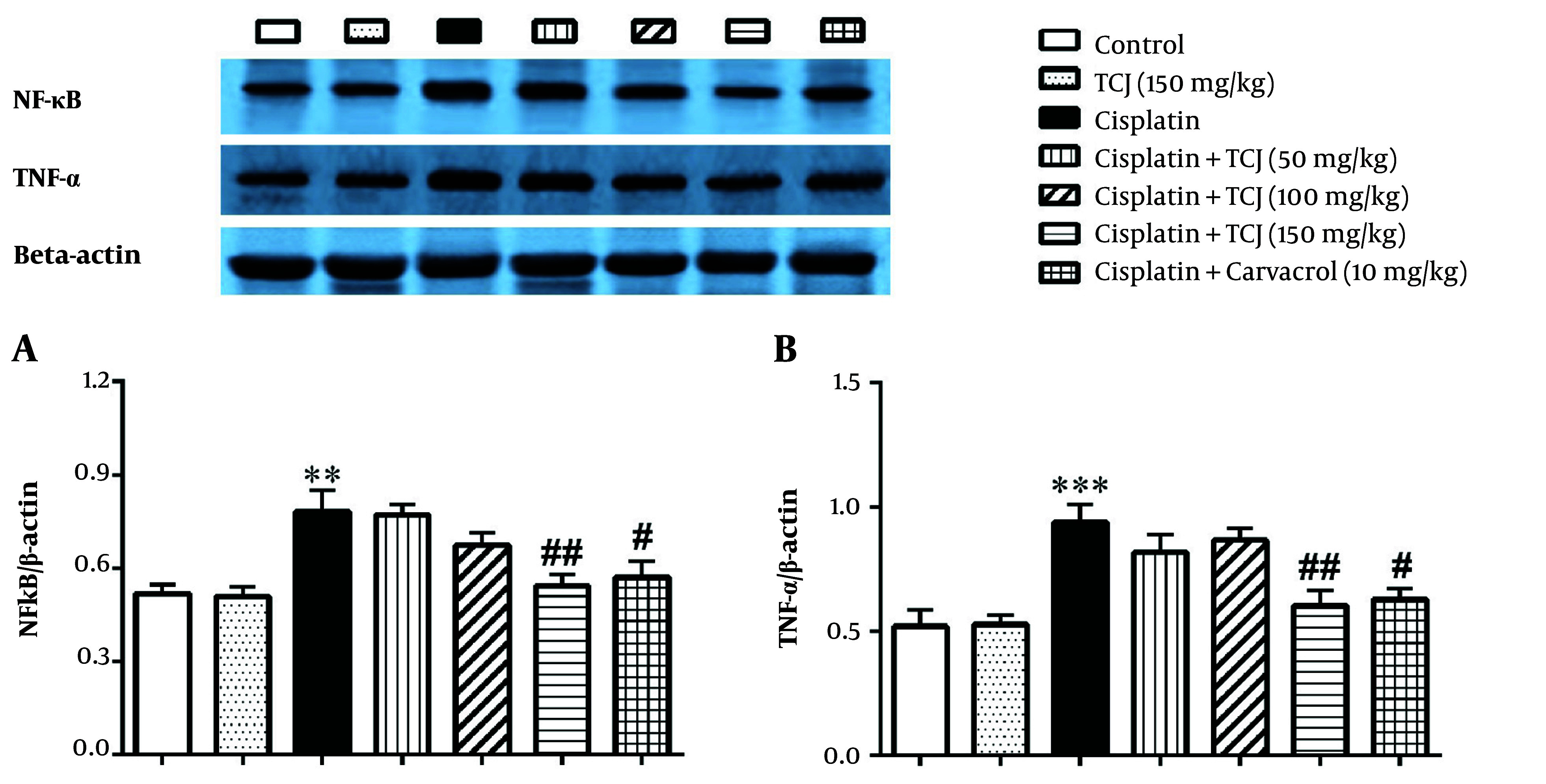
The immunoblotting analysis of the nuclear factor kappa-B (NF-κB) and tumor necrosis factor-alpha (TNF-α protein) expression levels in the kidney tissues at the end of the experiment. Each value is presented as mean ± SD. n = 6/group. ٭٭P < 0.01, and ٭٭٭P < 0.001 compared to the control. #P < 0.05, and ##P < 0.01 compared to the cisplatin group. TCJ: *Thymus caramanicus* jalas.

Our results also showed that cisplatin significantly increased the NF-κB (P < 0.01) and TNF-α (P < 0.001) protein expression levels, two inflammatory markers, in the renal tissue of the cisplatin-administered mice compared to the control group (Figure 3). *Thymus Caramanicus* Jalas (150 mg/kg) significantly decreased NF-κB and TNF-α protein expression levels in the renal tissue of the cisplatin + TCJ (150 mg/kg) group compared to the cisplatin group (P < 0.01, Figure 3). Additionally, carvacrol at a dose of 10 mg/kg significantly decreased NF-κB and TNF-α protein expression levels in the kidney tissue of the cisplatin + carvacrol (10 mg/kg) group compared to the cisplatin-administered mice (P < 0.05, Figure 3). 

### 4.5. The Effect of Thymus Caramanicus Jalas and Carvacrol on Kidney Tissues Apoptosis

**Figure 4.**
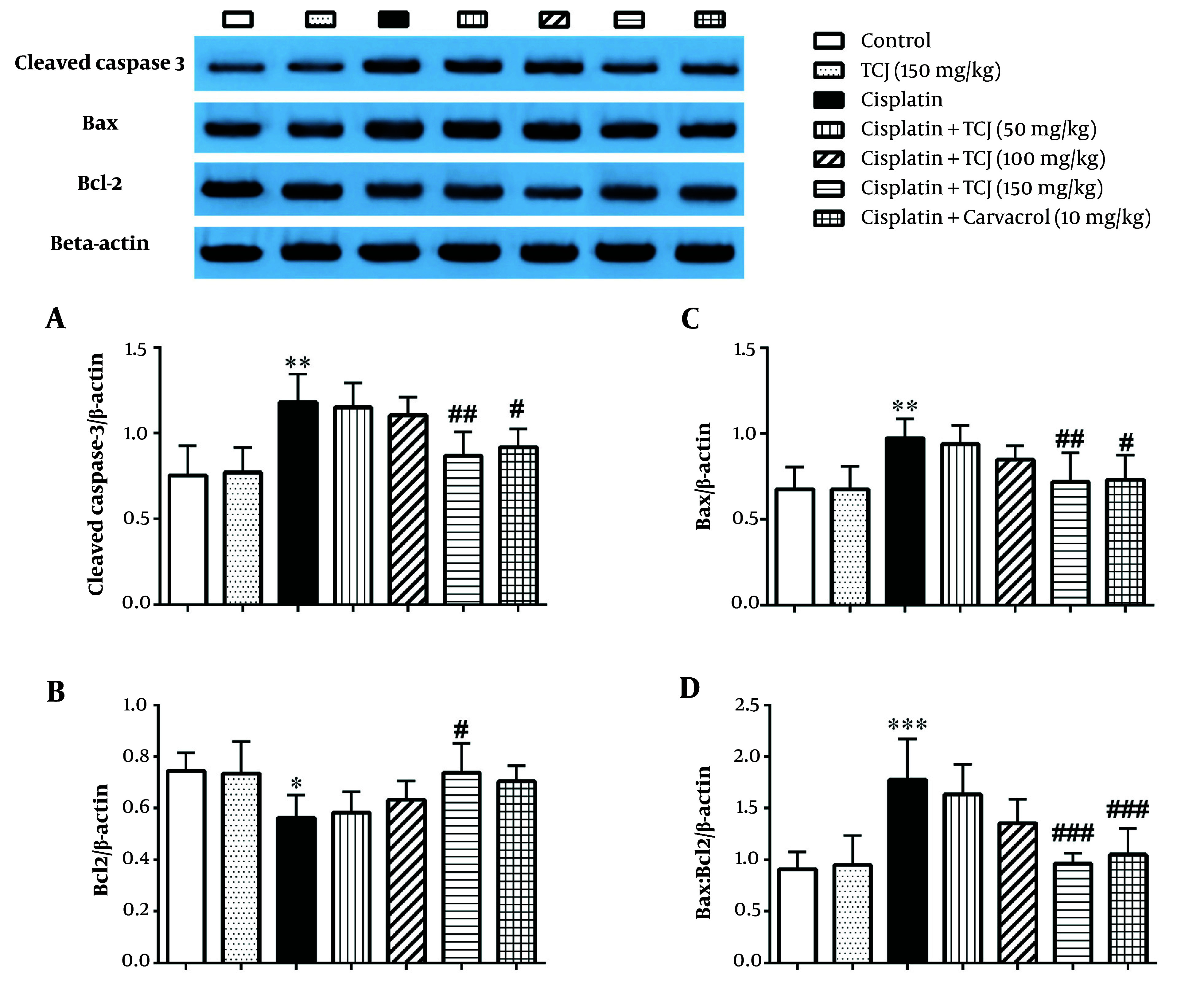
The Immunoblot analysis of the cleaved caspase-3 (A), Bax; (B), Bcl-2; (C), Bax/Bcl-2 ratio; (D) proteins in the renal tissues at the end of the experiment. Each value is shown as mean ± SD. n = 6/group. ٭P < 0.05, ٭٭P < 0.01, and ٭٭٭P < 0.001 compared to the control. #P < 0.05, ##P < 0.01, and ###P < 0.001 compared to the cisplatin group. TCJ, *Thymus caramanicus* jalas.

Cisplatin significantly increased cleaved caspase-3 (P < 0.01) and Bax (P < 0.01) protein expression levels, while it decreased Bcl-2 protein expression levels (P < 0.05) in the renal tissue of the cisplatin-administered mice compared to the control group (Figure 4A-C). Furthermore, TCJ (150 mg/kg) and carvacrol (10 mg/kg) ameliorated cisplatin-induced cleaved caspase-3 (P < 0.01 and P < 0.05, respectively) and Bax (P < 0.01 and P < 0.05, respectively) protein expression levels in the renal tissue of the cisplatin + TCJ (150 mg/kg) and cisplatin + carvacrol (10 mg/kg) groups compared to the cisplatin-administered mice (Figure 4A - B). Although TCJ (150 mg/kg) increased Bcl-2 protein expression (P < 0.05) levels in the renal tissue of the cisplatin + TCJ (150 mg/kg) group compared to the cisplatin group (Figure 4C), carvacrol (10 mg/kg) did not increase Bcl-2 protein expression in renal tissue (P > 0.05) (Figure 4C). 

As shown in Figure 4D, TCJ (150 mg/kg) and carvacrol (10 mg/kg) significantly decreased the Bax:Bcl-2 ratio in the renal tissue of the cisplatin + TCJ (150 mg/kg) and cisplatin + carvacrol (10 mg/kg) groups compared to the cisplatin group (P < 0.001).

### 4.6. The Effect of Thymus Caramanicus Jalas and Carvacrol on Kidney Tissues Damage Score

**Figure 5.**
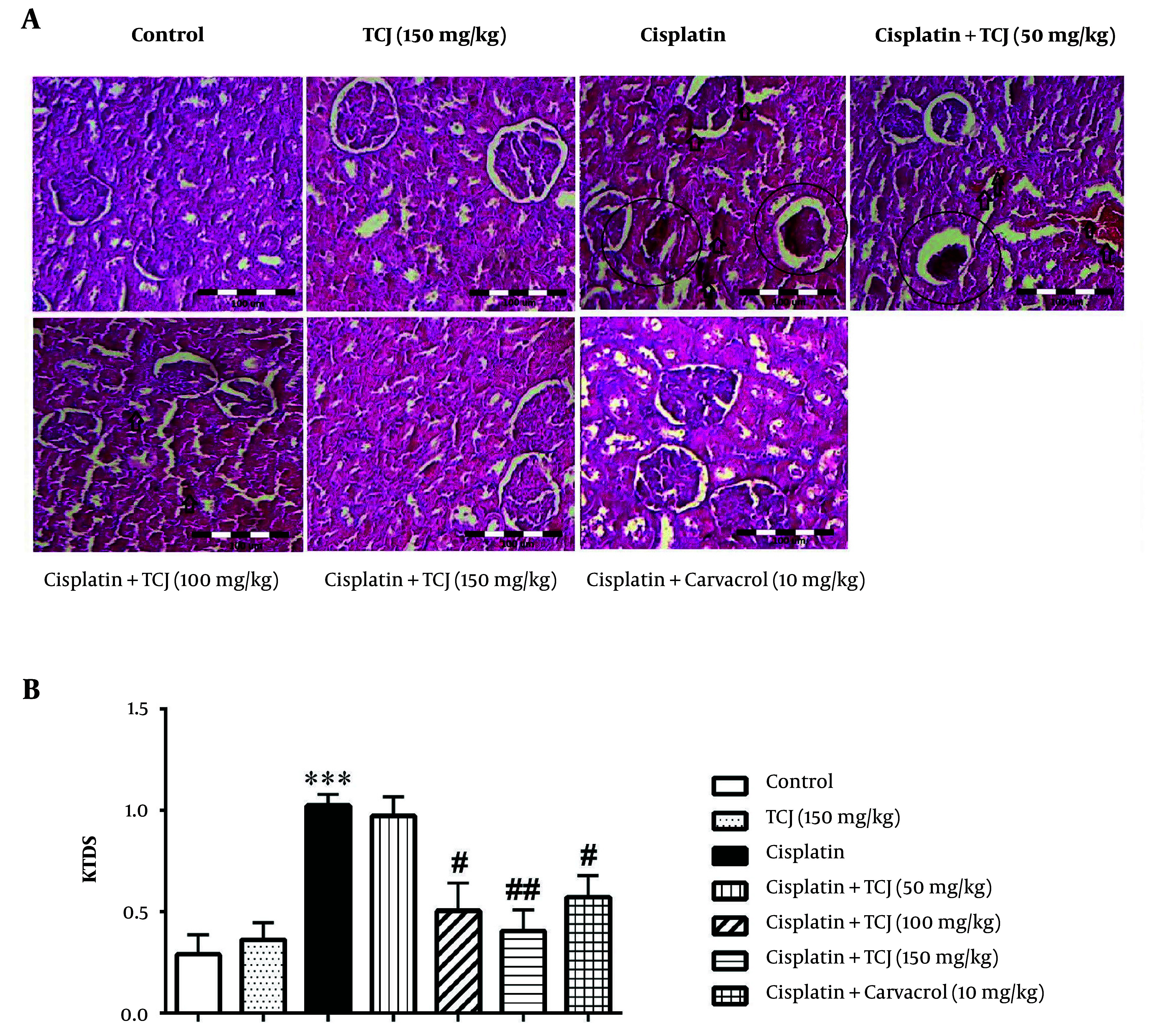
A, hematoxylin and eosin stained sections (magnification 100 ×) in the renal tissues at the end of the experiment. Score 0 - 0.5 was considered as normal and scores 1 - 3 were considered as mild, moderate, and severe damage depending on the presence of leukocyte infiltration, glomerular atrophy, and tubular necrosis in the renal tissues. Circle: Glomerular atrophy/tubular necrosis; arrow: Leukocyte infiltration; B, KTDS in all groups at the end of the experiment. Each value is shown as mean ± SD. n = 6/group. ٭٭٭P < 0.001 compared to the control. #P < 0.05, and ##P < 0.01 compared to the cisplatin group. TCJ, *Thymus caramanicus* jalas; KTDS, kidney tissue damage score.

Based on the hematoxylin and eosin results, no pathological conditions were detected in the renal tissue of the control group (Figure 5A). However, the cisplatin-administered mice showed significant kidney tissue damage compared to the control group (P < 0.001, Figure 5B). *Thymus Caramanicus* Jalas (100 and 150 mg/kg) ameliorated the kidney tissue damage caused by cisplatin, as evidenced by significantly decreased KTDS in the cisplatin + TCJ (100 mg/kg) and cisplatin + TCJ (150 mg/kg) treated mice compared to the cisplatin-administered mice (P < 0.05 and P < 0.01, respectively, Figure 5B). Additionally, carvacrol (10 mg/kg) significantly decreased the KTDS in the cisplatin + carvacrol (10 mg/kg) group compared to the cisplatin group (P < 0.05, Figure 5B). 

## 5. Discussion

In this study, we tested the effects of TCJ extract and its main component carvacrol on cisplatin-induced nephrotoxicity in mice. While cisplatin is beneficial in cancer treatment, it can lead to kidney damage depending on the dosage (29). Our results showed that cisplatin elevates serum Cr and BUN levels in the cisplatin-administered mice (Figure 2). However, TCJ extract mitigated these effects in mice receiving both TCJ extract and cisplatin (Figure 2). Carvacrol, the primary compound in TCJ herb, also showed a similar protective effect on serum Cr and BUN levels in mice treated with carvacrol and cisplatin (Figure 2). Furthermore, previous studies by Tezcan et al. (30) and Ozturka et al. (31) support these findings, highlighting cisplatin-induced renal dysfunction and the potential of carvacrol to reduce BUN and creatinine levels in an animal model with renal ischemia-reperfusion.

Our results also showed that cisplatin increases lipid peroxidation (MDA concentration) in the kidney tissue of administered mice. Nevertheless, TCJ extract (150 mg/kg) ameliorated the MDA level in the kidney tissue of TCJ-treated mice that received cisplatin (Table 1). Additionally, our study showed that cisplatin severely weakens the kidneys' antioxidant system, leading to decreased TAC levels, GSH content, and SOD, catalase, and GPx activity levels in renal tissue (Table 1). It has been shown that cisplatin increases ROS levels in the kidney, causing lipid peroxidation and weakening the antioxidant system (32). This condition causes oxidative stress in the kidneys and leads to kidney damage.

On the other hand, Honari et al. showed that TCJ and its compounds have antioxidative effects via the scavenging of free radicals in the diabetes mellitus model (33). *Thymus Caramanicus* Jalas increases SOD and catalase enzyme activity, two important components of the antioxidant system, and decreases lipid peroxidation in this model (33). Consistent with this study, our observations indicate that TCJ extract significantly elevates TAC levels, GSH content, and catalase, GPx, and SOD activity levels in the renal tissue of TCJ-treated mice that received cisplatin (Table 1). Additionally, carvacrol can increase GPx and SOD activity levels in the renal tissue of carvacrol-treated mice receiving cisplatin (Table 1). However, carvacrol did not alter catalase, TAC, and MDA indicators in carvacrol-treated mice that received cisplatin. This was consistent with the research of Safaei et al., which showed that the subfractions of TCJ and its main component (carvacrol) have radical-scavenging activity (15).

Interestingly, they reported that the whole herb essential oil showed better antioxidant function compared to carvacrol, its main constituent (15). The presence of components other than carvacrol, such as apigenin, luteolin, rosmarinic acid, flavonoids, and ferulic acid, has a synergistic effect on the total antioxidant properties of TCJ herb (15, 34, 35). Our study also showed that cisplatin increases inflammation markers, such as NF-κB and TNF-α proteins, in kidney tissue (Figure 3). Moreover, TCJ extract (150 mg/kg) and carvacrol can reduce the expression of these proteins involved in the inflammatory process (Figure 3). In this regard, Ramesh and Reeves reported that cisplatin induces nephrotoxicity by increasing NF-κB and TNF-α as inflammatory proteins in the kidney (36). Additionally, TNF-α induces the expression of other inflammatory cytokines in kidney tissue (37). Furthermore, oxidative stress can increase the production of NF-κB and TNF-α proteins (37).

In our study, cisplatin increased the expression levels of cleaved caspase-3 and Bax proteins and decreased the expression levels of Bcl-2 protein in the renal tissue of cisplatin-treated mice (Figure 4A-C). Moreover, our study showed that TCJ (150 mg/kg) and carvacrol (10 mg/kg) could ameliorate the cisplatin-induced expression of cleaved caspase-3 and Bax proteins in renal tissue (Figure 4A-B). *Thymus Caramanicus* Jalas also increased the Bcl-2 protein expression level in the renal tissue of cisplatin-administered mice (Figure 4C). However, 10 mg/kg of carvacrol did not increase Bcl-2 protein expression (Figure 4C). In line with the present study, Hajializadeh et al. showed that TCJ extract can ameliorate high glucose-induced neural apoptosis in in vitro and in vivo models by decreasing activated caspase 3, cytochrome c release, and Bax protein expression levels (14). Furthermore, our previous study showed that carvacrol has a protective effect against paracetamol-induced renal apoptosis at a dose of 10 mg/kg by decreasing cleaved caspase 3 and Bax protein expression levels (21).

Our results also showed that cisplatin increases kidney tissue damage (Figure 5). Although TCJ extract and carvacrol could attenuate the kidney tissue damage induced by cisplatin (Figure 5), it has been shown that cisplatin increases acute tubular necrosis and can lead to kidney damage (30). In this regard, it is known that cisplatin eventually causes kidney damage via renal tubular necrosis and apoptosis (38). It is possible that TCJ, through its main components, can reduce tissue damage by reducing the expression of proteins in the inflammatory pathway and inhibiting oxidative stress. In the present study, animals were treated with TCJ or carvacrol both before and after receiving cisplatin. This study focused on the therapeutic effects of TCJ compared to carvacrol (as its main constituent) in mice receiving cisplatin. However, one of the study's limitations is that it did not examine the treatment administered just before or just after receiving cisplatin. It is suggested to consider this point in future studies.

### 5.1. Conclusions

Based on our study, TCJ (150 mg/kg) and its main component (carvacrol) exhibit antioxidant, anti-inflammatory, and anti-apoptotic effects in the renal tissue of cisplatin-administered mice. Moreover, TCJ and carvacrol can improve kidney function in cisplatin-treated mice. *Thymus Caramanicus* Jalas extract appears to reduce kidney damage, likely through its antioxidative, anti-inflammatory, and anti-apoptotic effects in this model. Therefore, TCJ extract and its components may be used in the future to reduce renal damage after cisplatin administration. However, further research is needed.

## Data Availability

The dataset presented in the study is available on request from the corresponding author during submission or after publication.
